# Multi-biofluid metabolomics analysis of allergic respiratory rhinitis and asthma in early childhood

**DOI:** 10.1016/j.waojou.2024.101013

**Published:** 2024-12-19

**Authors:** Chih-Yung Chiu, Meng-Han Chiang, Chieh-Ni Kuo, Mei-Ling Cheng, Gigin Lin

**Affiliations:** a Division of Pediatric Pulmonology, Department of Pediatrics, Chang Gung Memorial Hospital at Linkou, and Chang Gung University, Taoyuan, Taiwan; b Clinical Metabolomics Core Laboratory, Chang Gung Memorial Hospital at Linkou, Taoyuan, Taiwan; c Department of Biomedical Sciences, and Metabolomics Core Laboratory, Healthy Aging Research Center, College of Medicine, Chang Gung University, Taoyuan, Taiwan; d Department of Medical Imaging and Intervention, Imaging Core Laboratory, Institute for Radiological Research, and Clinical Metabolomics Core Laboratory, Chang Gung Memorial Hospital at Linkou, College of Medicine, Chang Gung University, Taoyuan, Taiwan

**Keywords:** Asthma, Children, Metabolomics, Multi-biofluid, Rhinitis, Short-chain fatty acid

## Abstract

**Background:**

Childhood rhinitis and asthma are allergic respiratory diseases triggered by common allergens, but they affect different parts of the respiratory system, leading to distinct symptoms. However, a comprehensive multi-biofluid metabolomics-based approach to uncover valuable insights into childhood allergies and allergen sensitization remains unaddressed.

**Methods:**

Seventy-six children, comprising 26 with rhinitis, 26 with asthma, and 24 healthy controls, were enrolled. Fecal, blood, and urine metabolomic analyses using ^1^H nuclear magnetic resonance (NMR) spectroscopy were conducted. An integrative analysis of their associations with allergen-specific IgE levels in the context of allergic rhinitis and asthma were also assessed.

**Results:**

The analysis of 228 various biofluid samples revealed strong positive correlations between stool and blood metabolites, while blood metabolites exhibited negative correlations with most urine metabolites. Five and 19 metabolites were significantly different in children with rhinitis and asthma, respectively (*P* < 0.05). Among them, blood isovaleric acid correlated positively with stool IgE levels in rhinitis, while stool butyric acid and acetic acid in asthma exhibited strong negative correlations with total serum and mite allergen-specific IgE levels (*P* < 0.01). Blood metabolic profiles appeared to have the highest area under the curve (AUC) of 0.732 for rhinitis, whereas stool metabolic profiles had the highest AUC of 0.799 for asthma.

**Conclusions:**

Multiple biofluid metabolomics provides comprehensive insights into childhood allergies, with blood profiles associated with allergic rhinitis and fecal profiles linked to asthma. Their short-chain fatty acid metabolites related to IgE levels emphasize the significant role of the gut microbiota in childhood rhinitis and asthma.

## Introduction

Childhood rhinitis and asthma are both allergic respiratory diseases associated with an inflammatory process despite the fact that rhinitis mainly affects the nose and upper respiratory tract, while asthma primarily affects the lower respiratory tract, leading to different symptoms.^1^ Allergens can serve as shared triggers for both conditions, leading to the subsequent activation of immune responses, particularly IgE-mediated processes, and the release of inflammatory mediators.^2^ However, the varying clinical presentations of these conditions underscore the need to understand the complex interplay of allergic and inflammatory responses in childhood rhinitis and asthma, necessitating a personalized therapeutic approach for each condition.

Metabolomics, the comprehensive study of metabolites within a biological system, enables researchers to understand the dynamic metabolic profile of an organism under various physiological conditions. Metabolomics is now a powerful analytical approach applied in various aspects of human diseases, offering insights into the metabolic processes and biomarkers associated with these conditions.^3^ Clinically, metabolomics is increasingly being applied in allergies research to investigate the molecular mechanisms involved in allergic reactions.^4^ Different metabolites in the human body fluids correspond to diverse physiological processes and are generated in specific locations within various organs and tissues. A comprehensive, multi-biofluid metabolomics-based approach to allergen sensitization associated with childhood allergies may help identify the pathological conditions affecting different phenotypes and reveal metabolites that could be important for clinical applications.^5^ However, the simultaneous translation of these findings into effective clinical applications has not been approached.

Nuclear magnetic resonance (NMR) spectroscopy is a principal analytical technique employed in metabolomics research, valued for its high reproducibility and ability to provide high-throughput molecular identification within biological fluids.^6^ The aim of this study was to simultaneously determine the metabolic profiles of multiple biofluids, including stool, blood, and urine, using ^1^H NMR spectroscopy in children with rhinitis or asthma. Apart from performing mutual correlation analysis between the metabolic profiles detected in each specimen, the relationships among these metabolites concerning allergen sensitization and childhood allergies, specifically rhinitis and asthma, were simultaneously analyzed. The metabolic profiles detected in these different biofluids were also analyzed for distinguishing childhood rhinitis and asthma.

## Materials and methods

### Study population

A cross-sectional case-control study was conducted from 2020 to 2023 to investigate the metabolic profiles in 3 different biofluids (stool, blood, and urine) of children aged 5 years diagnosed with asthma alone or rhinitis alone, as well as healthy controls. All enrolled participants were from northern Taiwan, sharing the same geographical region and ethnic group, ensuring a homogeneous sample population for more accurate and reliable research outcomes. Allergic airway diseases, including asthma based on the guidelines of the Global Initiative for Asthma (GINA)^7^ and the Allergic Rhinitis and its Impact on Asthma (ARIA) guideline for rhinitis,^8^ were physician-diagnosed using the same diagnostic criteria by the same pediatric pulmonologist at the outpatient clinics. Children who met the diagnostic criteria for rhinitis and asthma without antibiotics medications were included, whereas those with underlying airway anomalies, systemic diseases, or comorbid asthma or rhinitis were excluded from the study. Controls in the study consisted of healthy children with no prior history of atopic conditions. Data on demographics, family history of atopy, exposure to passive smoking, and household income with respect to atopic diseases was systematically collected. The Ethics Committee of Chang Gung Memorial Hospital approved this study under the reference number 202300319B0C501. Written informed consent was secured from the parents or guardians of all subjects in the study.

### Measurement of total serum, allergen-specific, and stool IgE levels

Egg and milk allergies are the most common food allergies in young Asian children, while *Dermatophagoides pteronyssinus* and *Dermatophagoides farinae* are the leading inhalant allergens, affecting over 95% of children.^9^ The total serum IgE level was assessed using the ImmunoCAP system (Phadia, Uppsala, Sweden), while specific IgE levels for allergens such as egg white, cow's milk, *D*. *pteronyssinus*, and *D*. *farinae* were determined through a commercially available IgE assay (ImmunoCAP Phadiatop Infant; Phadia). The stool IgE levels were determined by employing the Immunoglobulin E ELISA Kit (Immundiagnostik AG, Bensheim, Germany) in accordance with the manufacturer's guidelines.^10^

### Sample preparation

Spot stool, blood, and urine samples were collected in the morning from enrolled subjects and stored at −80° Celsius until required. The procedures for preparing different samples for spectrum acquisition have been detailed in previous publications.^10^^,^^11^ Briefly, 900 μL of stool supernatant or urine was mixed with 100 μL of 0.075 M phosphate buffer (pH 7.4) in deuterium water containing 0.1% 3-(trimethylsilyl)-propionic-2,2,3,3-d_4_ acid sodium salt (TSP). For plasma, 500 μL was mixed with 500 μL of phosphate buffer in deuterium water containing 0.08% TSP as an internal chemical shift reference standard. After centrifuging all samples at 12000g for 30 min at 4 °C, a 600 μL supernatant was then transferred to a 5-mm NMR tube for analysis.

### ^1^H NMR spectroscopy

^1^H NMR, or proton nuclear magnetic resonance, is a technique used to determine the structure of organic compounds by analyzing the magnetic environments of hydrogen atoms in a sample. Due to its high reproducibility, minimal sample preparation, and ability to test multiple samples under consistent conditions, ^1^H NMR is ideal for detecting metabolites in multiple biofluids from the same subject as in this study. To obtain a comprehensive view of overall metabolic molecular information, untargeted ^1^H-NMR-based metabolomics were chosen and NMR spectra were therefore obtained using a Bruker Avance 600 MHz spectrometer (Bruker, Karlsruhe, Germany) at the Chang Gung Memorial Hospital in Taiwan.^11^ Sixty-four scans were acquired, generating 64K data points on a computer, with a spectral width of 10,000 Hz (corresponding to 10 ppm) followed by a 1D ^1^H Carr–Purcell–Meiboom–Gill (CPMG) spin-echo experiment. Prior to zero-filled Fourier transformation, 1D ^1^H NMR spectra were processed with an exponential line broadening of 0.3 Hz. Subsequently, the obtained NMR spectra were manually adjusted for phase, baseline correction, and referenced to the chemical shift of TSP (at δ 0.0 ppm) using TopSpin 3.2 software from Bruker BioSpin in Rheinstetten, Germany.

### NMR data processing and analysis

NMRProcFlow online software version 1.4 was employed to process the raw ^1^H NMR spectra.^12^ Alignment of the ^1^H NMR spectra was carried out, and the spectra were subjected to bucketing using the intelligent bucketing and variable size bucketing methods.^13^ Buckets corresponding to metabolites were recognized using Chenomx NMR Suite 9.0 professional software from Chenomx Inc. in Edmonton, AB, Canada. Spectral variables were subjected to a generalized log transformation (glog), followed by mean-centering and Pareto scaling to identify metabolites contributing to the discrimination between groups.^14^ Partial least squares-discriminant analysis (PLS-DA) was conducted using the online resource MetaboAnalyst 5.0. Metabolites displaying a *P*-value <0.05 or a variable importance in projection (VIP) score ≥1.0 in group comparisons were singled out, and functional metabolic pathways were analyzed using the Kyoto Encyclopedia of Genes and Genomes database (KEGG).

### Statistical analysis

Baseline characteristics of children with rhinitis, asthma, and healthy controls were compared and analyzed using appropriate univariate parametric and non-parametric tests, as needed. Correlation coefficients between the metabolic compounds in 3 different biofluid, total serum and allergen-specific, and stool IgE levels were determined using Spearman's correlation test in R software (version 4.0) from Lucent Technologies in NJ, USA. Random Forests models were used to rank metabolic profiles independently and validate them through a 20-fold stratified cross-validation test with the Boruta feature selection algorithm and classification.^15^ The performance of the model was examined by receiver operating characteristic (ROC) curves constructed by pROC using fivefold cross-validation.^16^ The statistical analysis was conducted using IBM SPSS Statistics for Windows, version 20.0. All hypothesis tests were two-tailed, and significance was defined as a *P*-value <0.05.

## Results

### Population characteristics

A total of seventy-six subjects were enrolled in this study, comprising 26 children with rhinitis, 26 children with asthma, and 24 healthy controls. The comparisons of baseline characteristics among children with rhinitis, asthma, and healthy controls are presented in Table 1. There were statistically significant differences in stool and total serum IgE levels, and specific IgE levels to *D. pteronyssinus* and *D*. *farinae* but not food allergens among children with rhinitis, asthma and healthy controls (all *P* < 0.01).

**Table 1:** Baseline characteristics of 76 children with rhinitis, asthma and health controls enrolled in this study.

Characteristics	Control n = 24	Rhinitis n = 26	Asthma n = 26	*P*-value
Age, yr	4.9 ± 0.8	4.9 ± 0.5	4.9 ± 0.6	0.941
Sex, male	13 (54.2%)	15 (57.7%)	18 (69.2%)	0.517
Body mass index, kg/m^2^	15.02 ± 1.19	16.18 ± 2.49	15.19 ± 2.52	0.155
Maternal atopy	10 (41.7%)	12 (46.2%)	16 (69.6%)	0.121
Parental smoking	9 (37.5%)	11 (42.3%)	12 (46.2%)	0.825
Annual household income, NTD				
Low, ≤500,000	9 (37.5%)	9 (34.6%)	9 (36.0%)	0.498
Medium, 500,000-1000000	11 (45.8%)	9 (34.6%)	13 (52.0%)	
High, >1,000,000	4 (16.7%)	8 (30.8%)	3 (12.0%)	
IgE levels, kU/L				
Stool IgE	4.0 ± 8.3	16.8 ± 29.0	4.6 ± 3.5	**<0.001**
Total serum IgE	76.11 ± 145.07	269.29 ± 318.49	313.58 ± 393.30	**<0.001**
*D. p*-specific IgE	4.59 ± 20.32	27.80 ± 34.73	25.85 ± 30.20	**0.003**
*D. F*-specific IgE	3.81 ± 17.51	17.41 ± 25.63	17.57 ± 24.81	**0.001**
Egg white specific IgE	0.34 ± 0.74	0.69 ± 1.41	0.51 ± 0.69	0.096
Milk specific IgE	0.29 ± 0.51	0.49 ± 1.34	0.51 ± 0.83	0.432

Data shown are mean ± SD or number (%) of patients as appropriate. yr, year; NTD; New Taiwan dollar; IgE, immunoglobulin E; *D. p*, *Dermatophagoides pteronyssinus*; *D. f*, *Dermatophagoides farinae.* All *P*-values <0.05 among the 3 groups, which is in bold, are significant.

### Association between stool, blood, and urine metabolites

^1^H NMR data from a total of 228 samples of various biofluids were analyzed and buckets varied across NMR spectra, corresponding to known metabolites. The Spearman's rank correlation coefficients between stool, blood and urine metabolites are shown in Fig. S1. The stool metabolites were strongly positively correlated with blood metabolites, whereas blood metabolites were negatively correlated with most of the urine metabolites. Among them, stool fumaric acid and blood acetylcarnitine, 3-hydroxybutyric acid, methionine, and arabinose were positively and negatively correlated with blood and urine metabolites respectively. However, the same metabolites in these 3 different biofluids were not strongly correlated each other (Fig. S2).

### Identification of metabolites in different samples for rhinitis and asthma

Metabolites with significantly different expression levels (*P*-value <0.05 for the fold change) between children with rhinitis or asthma and controls for stool, blood, and urine samples are listed in Table 2. Compared with healthy controls, isovaleric acid and phenylalanine in blood and N,N-dimethylglycine, alanine, and chlophedianol in urine were found to be significantly different in children with rhinitis. In contrast, there were 6, 8, and 6 metabolites found to be significantly different in stool, blood, and urine samples respectively in children with asthma.

**Table 2:** The VIP score and fold change of metabolites significantly differentially expressed between children with rhinitis and asthma and controls in stool, blood, and urine samples.

Metabolites	Stool						Blood						Urine					
	Rhinitis			Asthma			Rhinitis			Asthma			Rhinitis			Asthma		
	VIP score	Fold change	*P*	VIP score	Fold change	*P*	VIP score	Fold change	*P*	VIP score	Fold change	*P*	VIP score	Fold change	*P*	VIP score	Fold change	*P*
Butyric acid	2.23	1.31	0.114	1.76	0.61	**0.006**	–	–	–	–	–	–	–	–	–	–	–	–
β-Alanine	1.53	0.68	0.435	2.30	2.04	**0.012**	–	–	–	–	–	–	–	–	–	–	–	–
Fumaric acid	0.45	1.21	0.293	1.82	0.60	**0.026**	3.68	1.02	0.962	1.44	1.34	0.631	–	–	–	–	–	–
Methylamine	1.75	1.14	0.097	0.28	0.80	**0.028**	–	–	–	–	–	–	–	–	–	–	–	–
Inosine	0.40	1.07	0.916	4.27	1.83	**0.031**	–	–	–	–	–	–	–	–	–	–	–	–
Acetic acid	0.67	1.02	0.370	1.02	0.76	**0.048**	1.39	0.72	0.293	0.87	0.84	0.660	0.37	0.97	0.698	0.75	0.92	0.378
Isovaleric acid	–	–	–	–	–	–	1.57	1.16	**0.006**	0.13	1.00	0.617	–	–	–	–	–	–
Phenylalanine	0.17	0.96	0.931	0.50	1.19	0.630	1.12	1.13	**0.046**	0.75	0.92	0.159	–	–	–	–	–	–
Acetylcarnitine	–	–	–	–	–	–	0.60	0.96	0.149	1.37	0.88	**0.003**	–	–	–	–	–	–
3-Hydroxybutyric acid	–	–	–	–	–	–	2.02	0.58	0.172	3.07	0.35	**0.008**	–	–	–	–	–	–
Succinic acid	0.82	0.58	0.693	1.00	0.74	0.096	0.01	1.08	0.242	1.33	0.84	**0.010**	0.54	0.69	0.486	0.81	0.73	0.234
Citric acid	–	–	–	–	–	–	0.87	0.94	0.144	1.28	0.89	**0.010**	0.38	0.73	0.713	1.12	0.70	**0.040**
Lysine	0.43	0.90	0.870	0.91	1.17	0.752	0.31	0.98	0.311	0.93	0.93	**0.021**	1.23	0.88	0.063	0.85	0.94	0.069
Glutamic acid	1.08	0.85	0.402	1.10	0.80	0.235	0.21	0.99	0.544	0.87	0.94	**0.023**	–	–	–	–	–	–
Ornithine	–	–	–	–	–	–	0.13	0.99	0.610	1.11	0.90	**0.029**	–	–	–	–	–	–
Asparagine	–	–	–	–	–	–	0.35	0.96	0.275	1.10	0.87	**0.048**	–	–	–	–	–	–
N,N-Dimethylglycine	0.35	0.95	0.855	0.43	0.78	0.829	–	–	–	–	–	–	2.28	0.61	**0.003**	1.62	0.66	**0.001**
Alanine	0.45	0.82	0.900	0.88	1.10	0.495	0.39	1.05	0.531	0.29	0.97	0.705	1.89	0.79	**0.012**	1.46	0.64	**0.003**
Chlophedianol	–	–	–	–	–	–	–	–	–	–	–	–	3.12	1.27	**0.012**	1.51	1.34	0.138
Fucose	–	–	–	–	–	–	–	–	–	–	–	–	1.50	0.79	0.117	1.79	0.69	**0.002**
Creatinine	–	–	–	–	–	–	0.49	1.03	0.596	0.01	0.98	0.992	0.04	1.00	0.113	0.04	1.00	**0.042**
Glycine	0.08	0.90	0.916	0.20	0.98	0.538	0.66	1.10	0.494	0.28	0.99	0.320	1.68	0.69	0.346	1.19	0.73	**0.047**

VIP, Variable Importance in Projection; PLS-DA, partial least squares-discriminant analysis. All *P*-values <0.05 between each of the 2 groups, rhinitis or asthma, and the controls, which is in bold, are significant.

### Association between metabolites and IgE levels for rhinitis and asthma

**Fig. 1:**
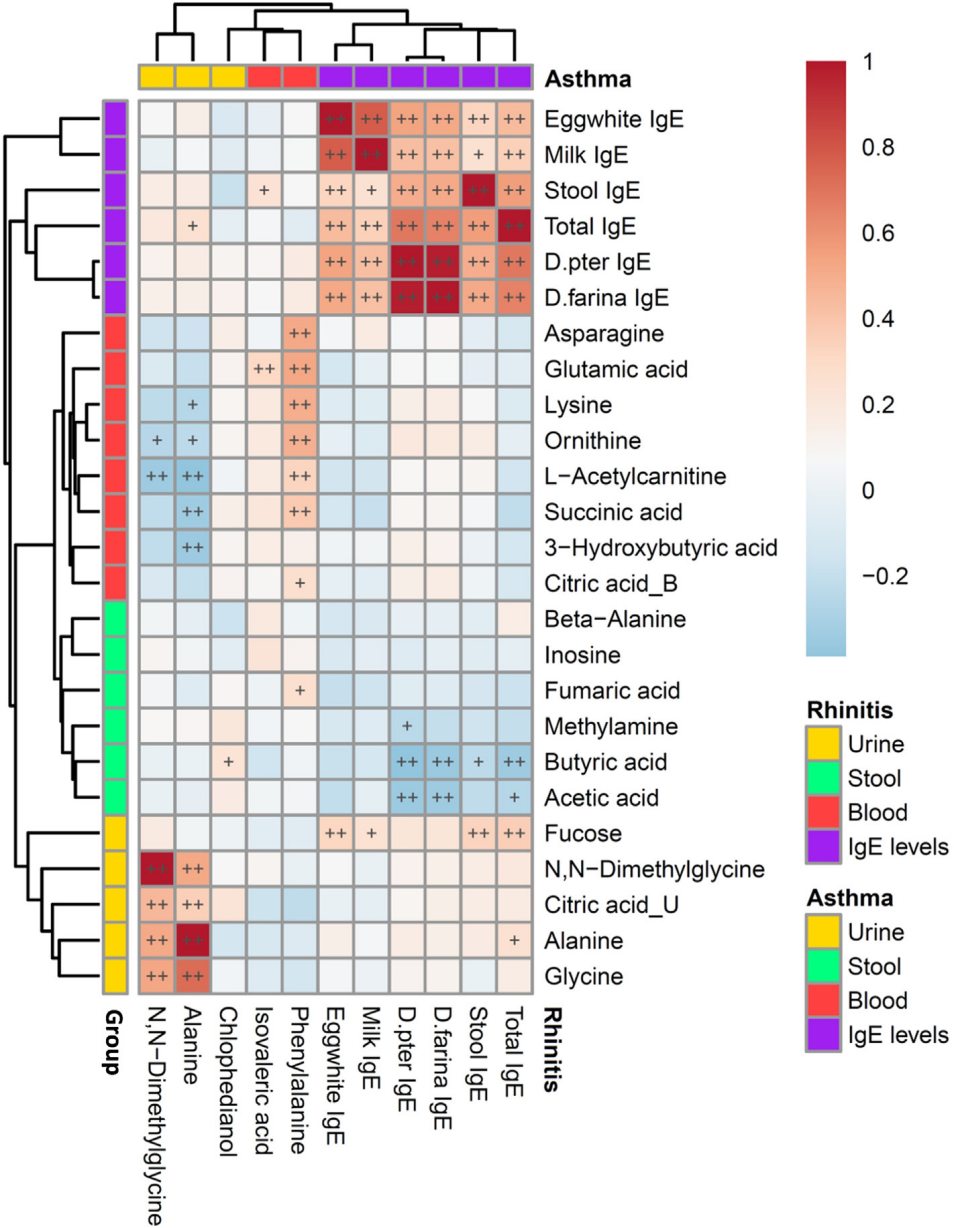
Heatmap of correlations between various biofluid metabolites significantly differentially expressed in children with rhinitis and asthma with total fecal IgE, total serum IgE, and allergen-specific IgE levels. Color intensity represents the magnitude of correlation. Red color represents positive correlations; blue color represents negative correlations. + symbol means a *P*-value <0.05; ++ symbol means a *P*-value <0.01

Stool IgE levels were significantly positively correlated with serum egg white-, milk-, *D. pteronyssinus*- and *D. farinae*-specific IgE levels (Fig. 1, all *P* < 0.01). Blood phenylalanine and urine alanine, related to rhinitis, were positively and negatively correlated with blood metabolites of acetylcarnitine and succinic acid, respectively, in association with asthma (all *P* < 0.01). Furthermore, blood isovaleric acid in children with rhinitis was positively correlated with stool IgE levels (*P* < 0.05). Moreover, stool butyric acid and acetic acid in asthmatic children were strongly negatively correlated with total serum and mite allergen-specific IgE levels, while urine fucose in asthmatic children was strongly positively associated with stool and serum total IgE levels (all *P* < 0.01).

### Metabolites that differentiate rhinitis and asthma from healthy controls

**Fig. 2:**
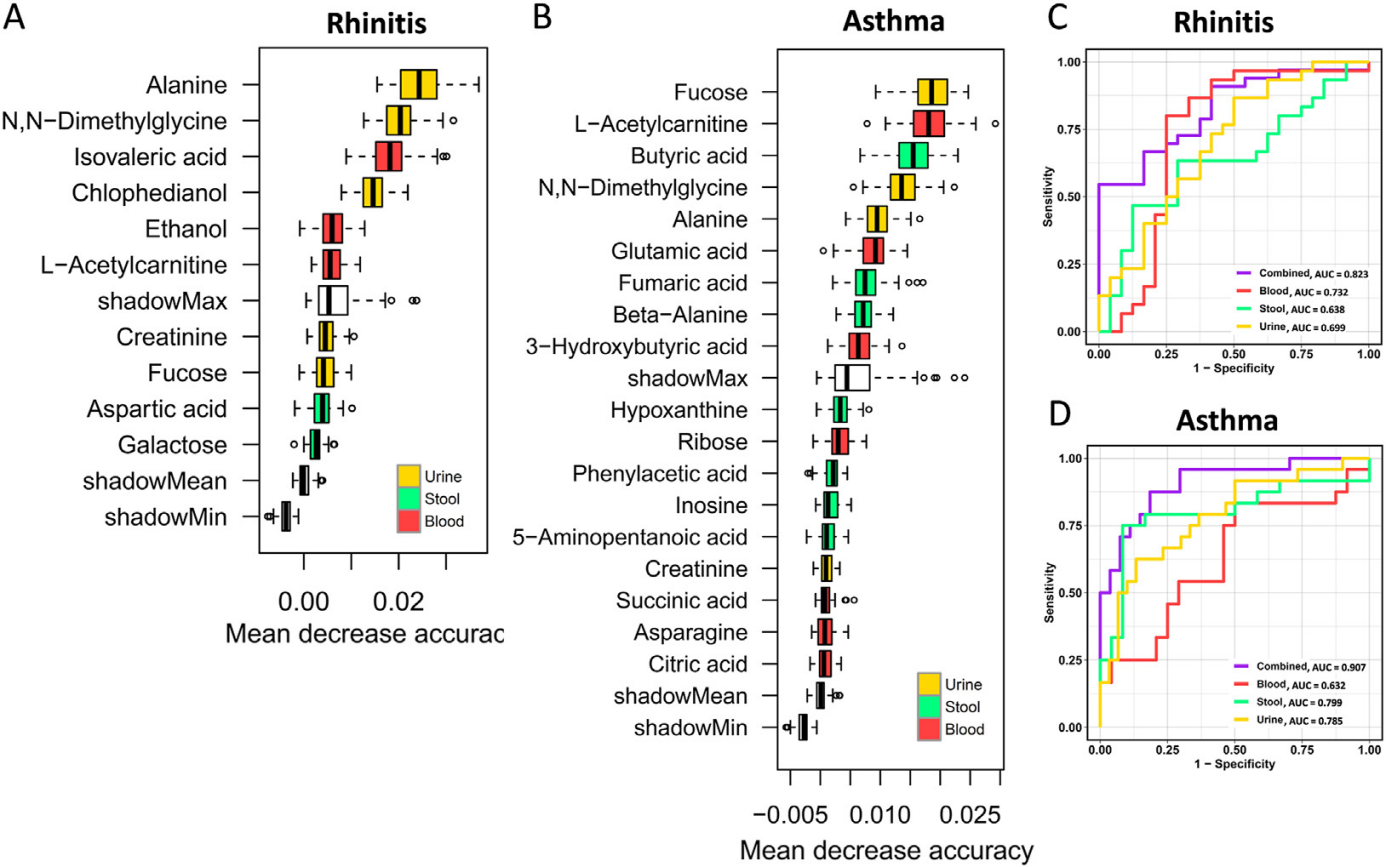
Random forests classification models based on a combined stool, blood, and urine metabolite markers for discriminating children with rhinitis (A) and asthma (B). Comparisons of ROC curve classification models among various biofluid metabolic profiles for discriminating children with rhinitis (C) and asthma (D). ROC, receiver operating characteristic; AUC, area under the curve

**Fig. 3:**
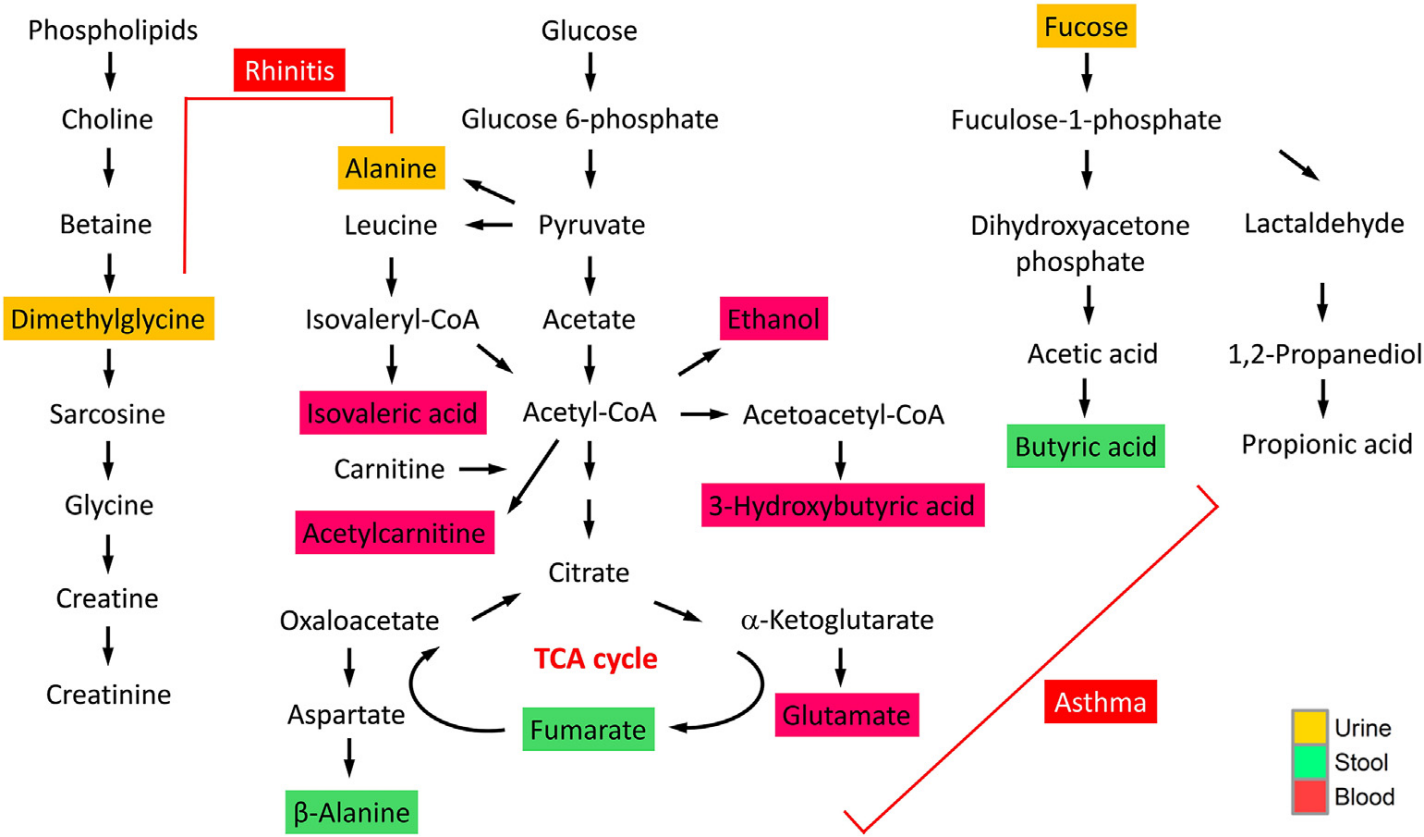
Schematic overview of metabolic pathways of significant and important metabolites associated with rhinitis and asthma. Yellow, urine samples; green, stool samples; red, blood samples

Random forests classification models based on stool, blood, and urine metabolites were performed to discriminate children with rhinitis (Fig. S3) and asthma (Fig. S4) from healthy controls. A combination of metabolites from these 3 biofluids including urine alanine, N,N-dimethylglycine, and chlophedianol, and blood isovaleric acid, ethanol, and acetylcarnitine were confirmed to have best importance for rhinitis (Fig. 2A). In contrast, urine fucose, N,N-dimethylglycine, and alanine, and blood acetylcarnitine, glutamate, and 3-hydroxybutyric acid, and stool butyrate, fumaric acid, and β-alanine were confirmed to have best importance for asthma (Fig. 2B). In assessing the classification efficiency among the 3 different biofluid models, the total area under the ROC curve (AUC) increased from 0.732 in the case of the blood model to 0.823 for the combined model for rhinitis (Fig. 2C). In contrast, for asthma, the AUC increased from 0.799 for the stool model to 0.907 for the combined model (Fig. 2D). Fig. 3 illustrates an integrated depiction of significant and important metabolites and their potential functional pathways for rhinitis and asthma.

## Discussion

Metabolomics has been extensively applied in allergies research, providing valuable insights into the complex metabolic alterations associated with allergic responses. However, a comprehensive multi-biofluid approach for obtaining molecular insights into allergic respiratory rhinitis and asthma has not been explored. This multiple biofluids (stool, blood, and urine) metabolomic analysis provides valuable insights into the molecular mechanisms related to allergic rhinitis and asthma in early childhood.

Nutrients and substances from the diet are absorbed into the bloodstream, with any unabsorbed components or metabolic byproducts being excreted in the stool, resulting in parallel representations in both blood and feces. The liver, a primary metabolic organ, processes numerous blood-borne metabolites, with some excreted in bile, entering the gut, and eventually eliminated in feces.^17^ The kidneys maintain stable metabolite concentrations in body fluids by adjusting excretion; when a metabolite concentration rises in the blood, the kidneys increase its excretion, leading to a decrease in urine metabolite concentration.^18^ These findings could particularly explain the positive correlation of metabolic profiles between stool and blood due to enterohepatic circulation, and a strong negative correlation between blood and urine due to renal excretion in this study.

Allergies entail diverse immune cell interactions and inflammation, which demands energy.^19^ In this study, rhinitis related metabolites were strongly associated with acetylcarnitine and succinic acid related to asthma. Acetylcarnitine is primarily involved in the transport of acetyl-CoA, a key molecule in energy metabolism that participates in the tricarboxylic acid cycle (TCA cycle), an essential process in cells for producing energy, and the oxidation of fatty acids.^20^ Allergic immune cell activity has been reported to involve lipid mediators like leukotrienes and prostaglandins in the regulation of inflammatory responses.^21^ This supports the idea that rhinitis and asthma, both allergic respiratory diseases, share a similar inflammatory process.

Analyzing the metabolomic pathways across multiple biofluids allows for a comprehensive understanding of the overall metabolic profile associated with a specific disease as in this study.^22^ Despite the use of metabolomics with different biofluids as a research approach to investigate rhinitis,^23^^,^^24^ the blood metabolic profile appeared to most effectively represent the association with allergic rhinitis in this study. Blood isovaleric acid, a short-chain fatty acid, can be produced by the gut microbiota and is also a part of the metabolism of branched-chain amino acids (BCAAs), specifically originating from leucine. A correlation between isovaleric acid and stool IgE levels was observed in this study, further supporting recent reports of a strong connection between gut microbiota and allergic rhinitis.^25^

Asthma, a complex disease shaped by genetic and environmental factors, has been reported in numerous studies utilizing metabolomics for prediction, diagnosis, and personalized treatment.^26^ Urinary and fecal metabolomic profiling has provided a link between microbe-environment interactions in the development of childhood asthma.^10^^,^^27^ Nevertheless, in this study, the fecal metabolic profile seemed to most effectively demonstrate the association with asthma. Furthermore, stool butyric acid and acetic acid, which are related to fucose metabolism produced by the gut microbiota, were strongly correlated with mite-specific IgE levels. In children with asthma, an imbalanced gut microbiota may reduce butyrate production, consequently diminishing the functionality of the intestinal barrier for allergens, ultimately leading to subsequent allergic reactions and symptoms.^28^ These findings indicate the crucial role of gut microbial dysbiosis in the development of childhood asthma, as observed in several studies on this issue.^29^

A limitation of this study is its small sample size, which may result in insufficient statistical power for conducting subanalyses. Although NMR spectroscopy is effective in providing highly reproducible metabolic fingerprints, it remains a relatively insensitive technique capable of detecting only metabolites present in high concentrations. To preserve the original state of subject samples, different solvents were not used to increase sensitivity and detect more metabolites, which may have resulted in incomplete biological information in this study. However, an age-matched group comparison analysis effectively minimizes the dissimilarities in metabolic compositions in this study. Most importantly, integrated multiple biofluid metabolomics analysis provides a more comprehensive insight into diseases.

In conclusion, multiple biofluid metabolomics offers the most comprehensive molecular insight into various aspects of health and disease. There is a positive correlation of metabolic profiles between stool and blood indicating enterohepatic circulation, but a strong negative correlation between blood and urine indicating renal excretion. A strong correlation of rhinitis-associated metabolites and asthma-related acetylcarnitine, which is involved in the oxidation of fatty acids for the regulation of inflammatory responses, supports the idea that rhinitis and asthma share a similar inflammatory process. The blood metabolic profile could most effectively represent the association with allergic rhinitis, while the fecal metabolic profile most effectively demonstrates the association with asthma. This finding indicates that distinct metabolic pathways reflect inflammation in allergic rhinitis, while the gut-lung axis primarily influences respiratory inflammation in asthma. Most importantly, short-chain fatty acids produced by the gut microbiota, including stool IgE-associated blood isovaleric acid related to rhinitis, and stool butyric acid associated with mite-specific IgE related to asthma, indicate a strong connection between the gut microbiota and childhood allergic rhinitis and asthma. However, additional research is necessary to explore the strength and mechanisms of these associations through functional studies.

## Abbreviations

AUC, area under the curve; KEGG, Kyoto Encyclopedia of Genes and Genomes; BCAA, branched-chain amino acid; CPMG, Carr–Purcell–Meiboom–Gill; glog, generalized log transformation; NMR, nuclear magnetic resonance; PLS-DA, partial least squares-discriminant analysis; ROC, receiver operating characteristic; TCA cycle, tricarboxylic acid cycle; TSP, 3-(trimethylsilyl)-propionic-2,2,3,3-d4 acid sodium salt; VIP, variable importance in projection.

## Funding

Chang Gung University, Taiwan; Health Aging Research Center, Taiwan; Chang Gung Memorial Hospital, Taiwan.

## Data availability

The datasets used and analyzed during this study are available from the corresponding author upon reasonable request. The data are not publicly available because of privacy restrictions.

## Author contributions

C.-Y.C., M.-L.C., and G.L. designed the study and drafted the manuscript. M.-H.C. performed experimental work and interpretation. C.-N.K. performed statistical analyses and presented the data. C.-Y.C. was responsible for clinical evaluation of the children and data collection. All authors discussed the results and approved the final draft for publication.

## Ethics approval

The study was conducted in accordance with the principles of the Declaration of Helsinki and approved by the Ethics Committee of Chang Gung Memorial Hospital (No. 202300319B0C501).

## Consent for publication

All authors have agreed with this publication in the World Allergy Organization Journal.

## Declaration of competing interest

All the authors declare no conflicts of interest in relation to the present study.
