# Supplementary material for: Multi-biofluid metabolomics analysis of allergic respiratory rhinitis and asthma in early childhood
Source: World Allergy Organ J. 2024 Dec 19;18(1):101013. doi: 10.1016/j.waojou.2024.101013 (PMC11731466; doi:10.1016/j.waojou.2024.101013)
Supplement: Multimedia component 1 [file mmc1.docx]

**Supplementary information**

**Multi-biofluid metabolomics analysis of allergic respiratory rhinitis and asthma in early childhood**

**
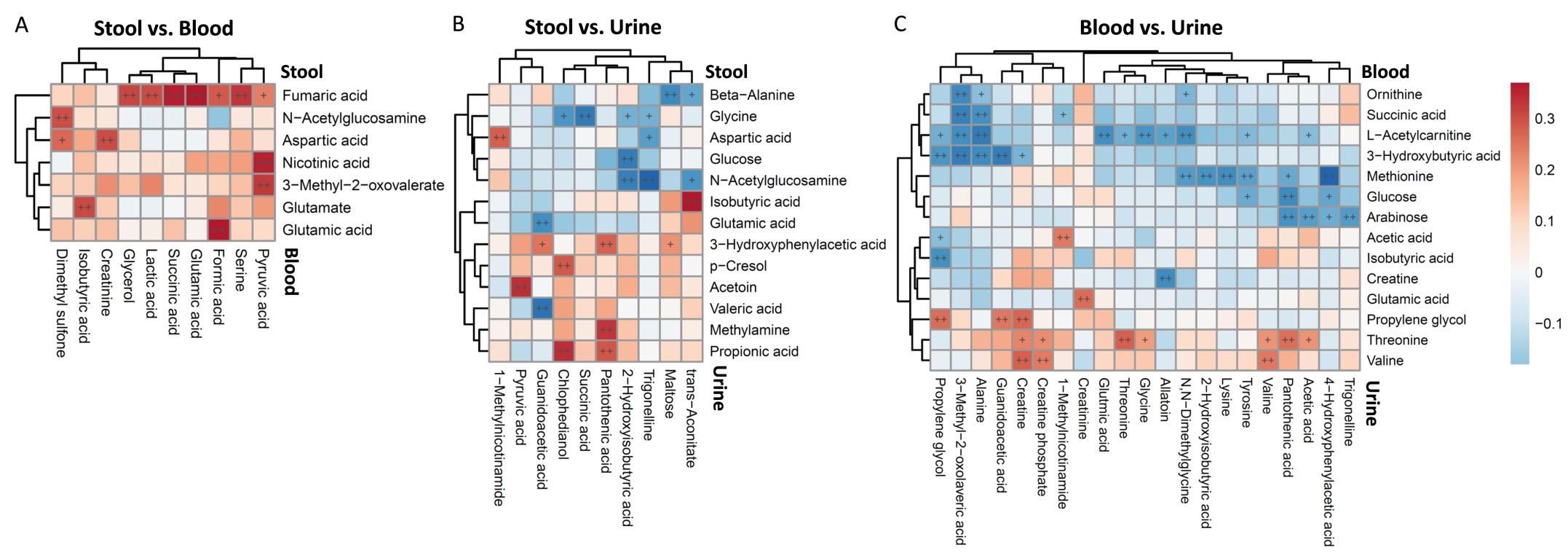
**

**Supplementary Figure S1.** Heatmaps of Spearman’s rank correlation coefficients of metabolites between stool and blood (A), between stool and urine (B), and between blood and urine (C). Color intensity represents the magnitude of correlation. Red, positive correlations; blue, negative correlations. + symbol means a *P*‐value < 0.05; ++ symbol means a *P*‐value < 0.01.

**
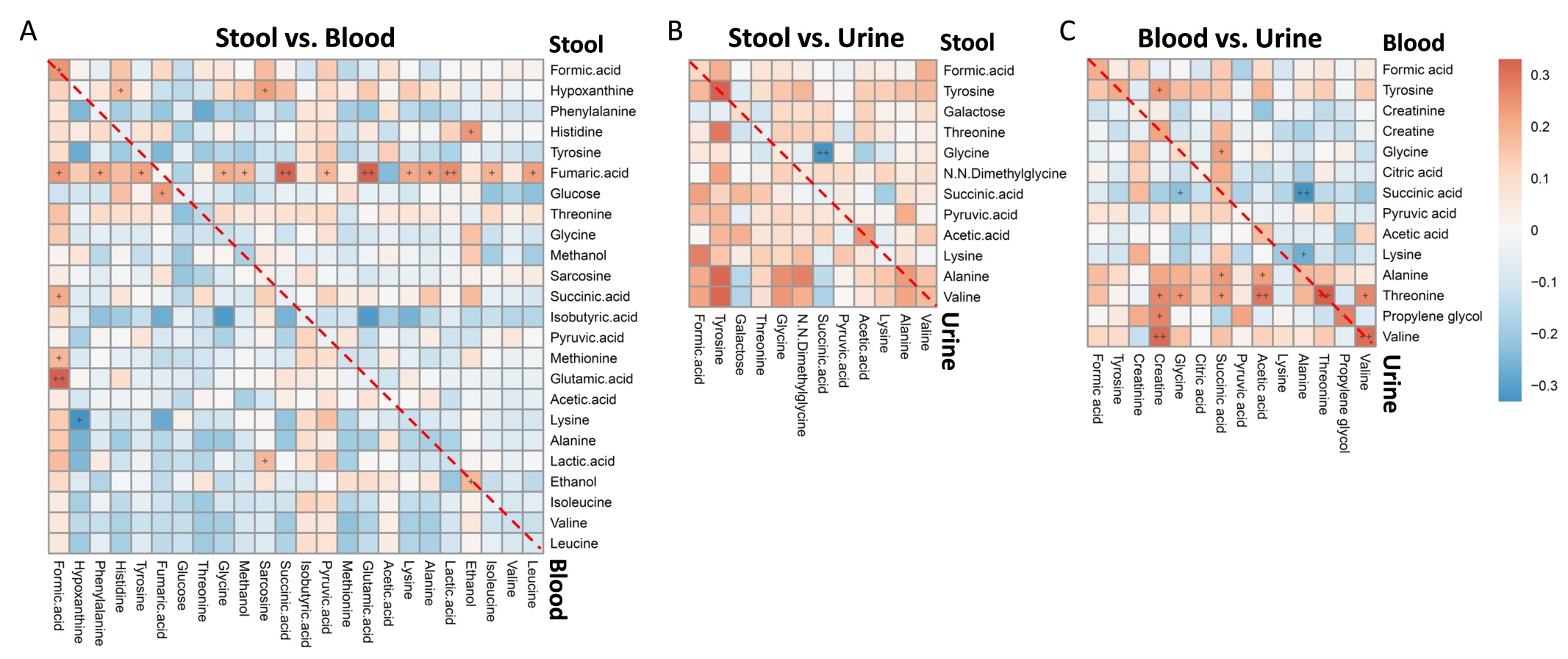
**

**Supplementary Figure S2.** Heatmaps of Spearman’s rank correlation coefficients of the same metabolites existed between stool and blood (A), between stool and urine (B), and between blood and urine (C). Color intensity represents the magnitude of correlation. Red, positive correlations; blue, negative correlations. + symbol means a *P*‐value < 0.05; ++ symbol means a *P*‐value < 0.01.


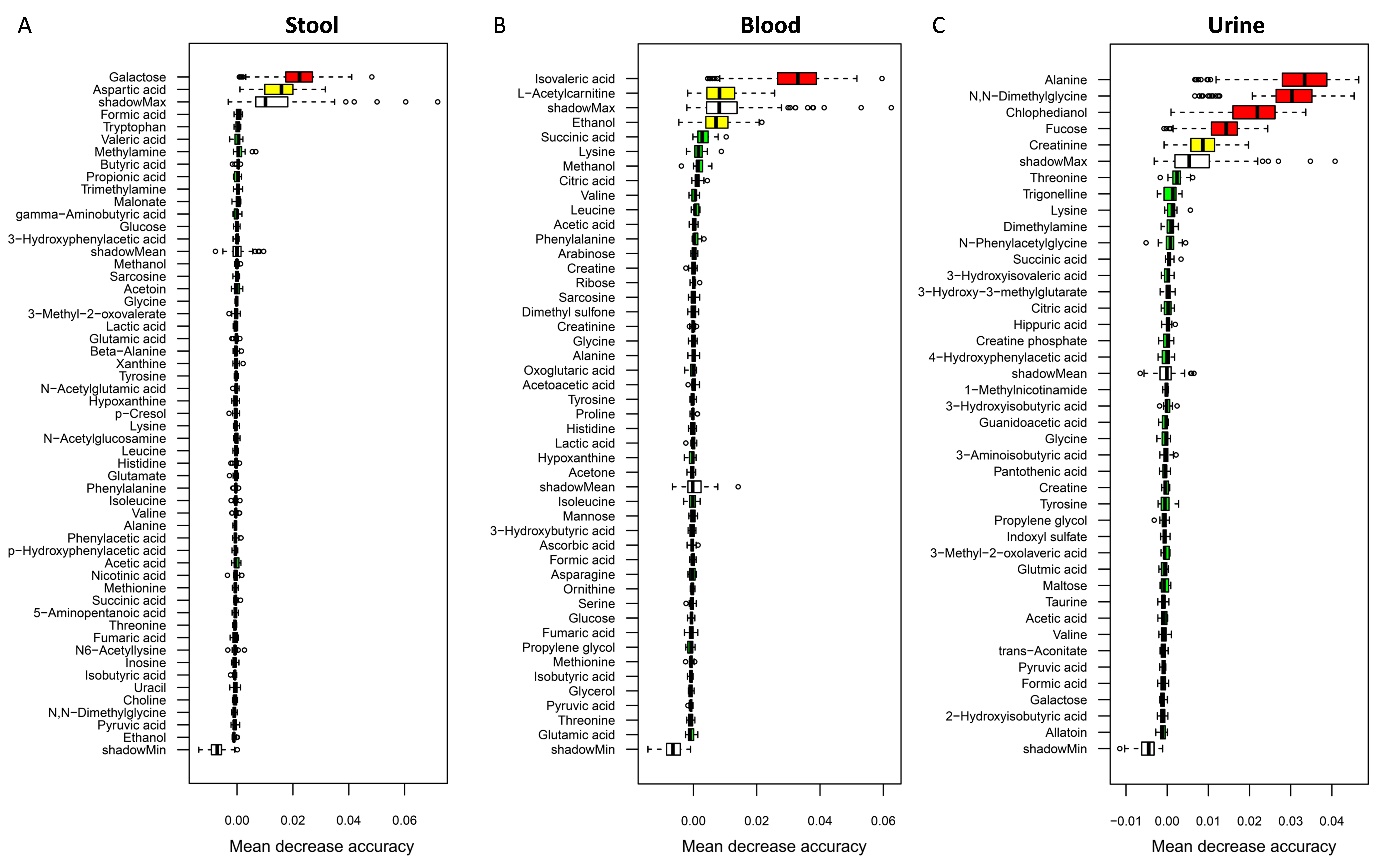


**Supplementary Figure S3.**

Markers for detecting children with rhinitis identified from metabolic profiles of stool (A), blood (B), and urine (C) using Random forests classification models. Markers are ranked in descending order of their importance to the accuracy of the model. The boxes represent 25th-75th percentiles, and black lines indicate the median.


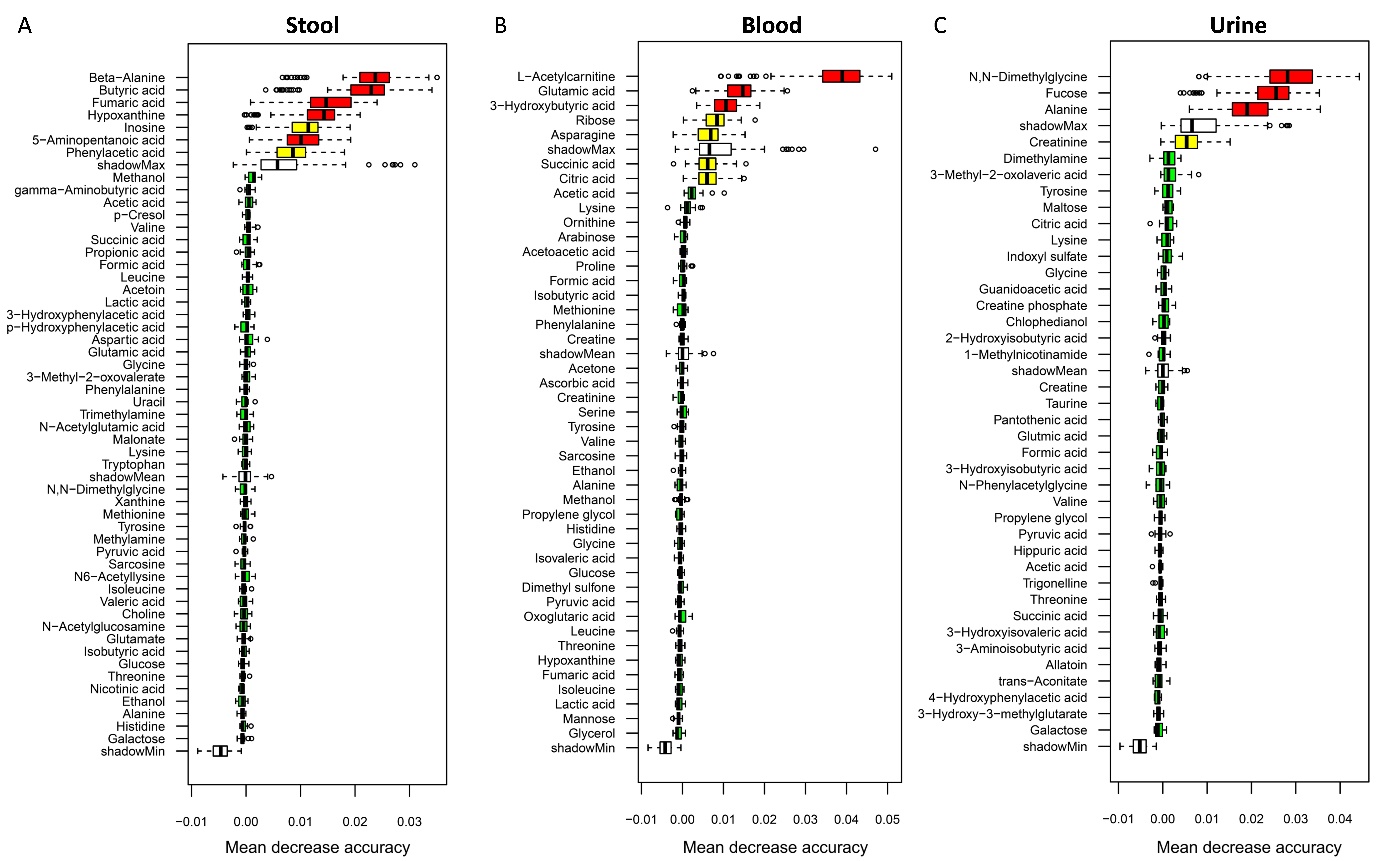


**Supplementary Figure S4.**

Markers for detecting children with asthma identified from metabolic profiles of stool (A), blood (B), and urine (C) using Random forests classification models. Markers are ranked in descending order of their importance to the accuracy of the model. The boxes represent 25th-75th percentiles, and black lines indicate the median.
